# 30 years of HIV: what have we learned?

**DOI:** 10.7448/IAS.17.4.19478

**Published:** 2014-11-02

**Authors:** Brian Gazzard

**Affiliations:** HIV Unit, Chelsea and Westminster Hospital, London, UK

## Abstract

I saw my first patient with severe immune deficiency in 1979 – a very low CD4 count had been noted, but it was not until the first reports of an epidemic occurred in 1981 that the correct diagnosis was made. Subsequently, I have seen more than 15,000 patients with HIV-related immune deficiency, and my life has changed from helping terminally ill patients to die with dignity, in the early part of the epidemic to now providing drugs for an eminently treatable condition – a true miracle. I have a number of observations about the epidemic. Firstly, the courage with which many young people faced death and disablement was truly awe inspiring, and was the chief reason many of the earlier doctors treating these patients stayed in the field. Secondly, the role of activists was overwhelmingly positive forcing the epidemic to the top of the scientific and political agenda and keeping it there. It is also important that activism helped move an ethical agenda reducing the stigma of HIV infection and producing a liberal legal framework which allowed testing and treatment to be acceptable. The right of the world population to health as espoused by Jonathan Mann and others is also crucial. Thirdly, the combination of academic research, activist pressure (and scientific input) and mammon in the form of the pharmaceutical industry acting in concert produced knowledge which led to effective treatment in a breathtakingly short time. Particular tribute in my mind needs to be paid to the pharmaceutical companies in this regard. I believe that the scientific achievements of HIV research illustrate two things. First, science builds from one generation to the next and most (but not all of us) need to be humble about our personal contribution. Second, HIV treatment illustrates the primacy of well conducted randomized control trials. While cohort studies can add to our detailed knowledge of the epidemic, randomised controlled trials remain the cornerstone of most major advances. Fourthly, when human beings act in concert towards a common goal, amazing things can be achieved. In the late 1990s, the possibility of treatment for the millions of people with HIV in the developing world was seen as a distant dream. The present situation whilst not perfect is a tribute to individuals, volunteers, government (particularly American government under President Bush) and personal philanthropy (the Bill & Melinda Gates Foundation, and the Clinton Foundation) that have used scientific knowledge to benefit the global population.

